# The Myval Balloon-Expandable Transcatheter Heart Valve Implant in Aortic and Mitral Interventions: A Single-Center Experience

**DOI:** 10.7759/cureus.80638

**Published:** 2025-03-15

**Authors:** Ramesh Patel, Gaurav K Mittal, Jai Bharat Sharma, Sanjay Gandhi, Dilip Jain

**Affiliations:** 1 Cardiology, Geetanjali Medical College and Hospital, Udaipur, IND; 2 Cardiothoracic and Vascular Surgery, Geetanjali Medical College and Hospital, Udaipur, IND

**Keywords:** aortic stenosis, balloon expandable transcatheter heart valves, myval, transcatheter aortic valve implant, transcatheter mitral valve in ring

## Abstract

Background:Balloon-expandable valve implants are widely used for percutaneous aortic and mitral valve replacement. This study presents our experience with the Myval implant (Meril Life Sciences, Vapi, India) in these positions.

Methods:This is a retrospective single-cohort observational study. Between March 2019 and August 2024, 15 patients underwent Myval implantation; out of them, 14 patients (93.33%) underwent transcatheter aortic valve implant (TAVI), and one underwent transcatheter mitral-valve-in-ring implant (TMViR). The mean age of our patients was 75.87±7.51 years (range: 64-90 years), with a slightly higher proportion of females (53.33%). All the patients were symptomatic and presented in New York Heart Association (NYHA) functional class II to IV. The mean EuroSCORE II was 7.99±5.64%, indicating more higher operative risk patients. Fourteen patients who underwent TAVI had severe aortic stenosis with varying degrees of regurgitation. The mean aortic annulus area in these patients was 390.20±74.49 mm², with a mean area-derived diameter of 22.19±2.17 mm. The most commonly used Myval implant sizes were 23 mm (33.33%) and 24.5 mm (33.33%). The procedures were conducted under deep conscious sedation unless general anesthesia was specifically necessary. All cases were performed through the femoral route, with three patients (20%) requiring a femoral arterial cut-down approach and the remaining 12 (80%) cases utilizing the percutaneous Seldinger technique, guided by an angiographic roadmap. Pre-ballooning was not mandatory and was required in only five of the TAVI cases and the TMViR case.

Results:In our study, no instances of valve migration, embolization, or deformation were reported. Coronary protection was required in four patients (28.6%) of TAVI procedures, while none required coronary stenting post-valve deployment. One patient underwent emergency coronary stenting under extracorporeal membrane oxygenation support before valve deployment as guide-induced left-main coronary dissection during a coronary protection procedure. Post-procedure, two patients had significant paravalvular leak, and two had residual stenosis against Myval, but both improved by post-ballooning with additional volume. The failure rate of the ProGlide percutaneous closure device was 13.3%. One patient had a navigator balloon rupture below the rated burst pressure, and one had a Python sheath tear during retrieval of the delivery system. No patients required permanent pacemakers (PPMs). The mean post-procedural hospital stay was 2.9 days. There were no procedural or 30-day mortalities. During a mean follow-up of 22 months, two patients (13.4%) died, one of them attributed to non-cardiac causes.

Conclusion: Our experience with Myval has shown it to be an effective and user-friendly option for both aortic and mitral interventions, demonstrating good procedural success rates and a favorable safety profile despite some minor concerns, making it a cost-effective choice for balloon-expandable transcatheter heart valves, particularly in developing countries like India.

## Introduction

Transcatheter aortic valve implantation (TAVI) has become the treatment of choice for elderly patients with severe calcific aortic stenosis (AS) who are at intermediate or high surgical risk, and it has now shown non-inferiority to surgical aortic valve replacement (SAVR) even in low surgical-risk patients [1-4]. The majority of TAVI procedures worldwide are performed using either a balloon-expandable SAPIEN-3 (Edwards Lifesciences, USA) or a self-expanding Evolut-R/Pro (Medtronic, USA) transcatheter heart valve (THV). Transcatheter mitral valve replacement (TMVR) using balloon-expandable THVs can be an alternative to open-heart surgery for patients with severe mitral valve disease after surgical valve replacement or annuloplasty ring repair or in native valves with severe mitral annular calcification who are not eligible for conventional surgery [5,6].

Meril Lifesciences, Gujarat, India, developed the Myval™ balloon-expandable THV. Following the Myval-1 study, the device was approved by the Central Drugs Standard Control Organization of India in October 2018 and CE marked in the European Community in April 2019 for transcatheter valve replacement [7].

In our tertiary care center, we have been performing transcatheter valve replacement procedures with self-expanding or balloon-expanding valves since 2019. This study presents our experiences with the Myval balloon-expandable valve for aortic and mitral positions.

## Materials and methods

Type of study and patient population

This is a retrospective single-cohort observational study. This study received approval from the Institutional Ethics Committee of Geetanjali Hospital, Udaipur (IEC//GMCH/2024/2013, dated August 24, 2024). Between March 2019 and August 2024, 15 patients received Myval implants at our tertiary care center. Of these, 14 patients underwent TAVI for the native aortic valve lesions, while one underwent TMVR in a fully rigid Edwards IMR Etlogix 24 mm mitral ring (Edwards Lifesciences). The surgical predictive operative mortality was assessed in every patient by EuroSCORE-II score, and patients were categorized as low (0-2%), intermediate (3-5%), or high-risk (>5%) [8,9]. The eligibility for the transcatheter procedure was finalized after a cardiac team evaluation and a detailed discussion with family members. All patients undergo a minimum of routine clinical examination, 12-lead electrocardiograms, 2D transthoracic or transesophageal echocardiogram, routine lab investigation, and computed tomography (CT) of the concerned area. We follow the American guidelines and standards in our clinical practice for eligibility criteria cut-off and to define the severity of valvular lesions [10-12].

Study objectives

This study was conducted to determine the pros and cons of the Myval balloon-expandable valve for aortic and mitral positions.

Data collection and statistical analysis

Patients' clinical profiles, laboratory, echocardiographic, and procedural data were collected from medical records. All data were analyzed using IBM SPSS Statistics for Windows, Version 19 (Released 2010; IBM Corp., Armonk, New York, United States). The data were presented as mean±standard deviation (SD) for parametric variables, median for nonparametric variables, and percentages or frequencies for categorical variables.

Imaging analysis and valve selection

The CT images using 3mensio advanced imaging were analyzed thoroughly to preplan every patient. The size of the Myval implant was decided in a heart team meeting in correlation with detailed CT measurements of the aortic root complex, left ventricular outflow tract (LVOT), coronaries, calcium dispersion, and femoral artery measurements. Meril Life Sciences, Vapi, India, developed a digital application to determine Myval size based on CT-measured 3D aortic annular area. For Myval size choice across the aortic annulus, we usually accept up to a maximum of 10-15% oversize in the tricuspid aortic and 5%-10% for the bicuspid aortic valve [13].

Outcome and follow-up

The minimum follow-up duration in our study was one month, and the mean follow-up duration was 22 months. Outcome variables were all-cause mortality, prosthetic valve dysfunction, stroke, myocardial infarction, and quality of life improvements in terms of the New York Heart Association (NYHA) functional class. Periprocedural outcome measures include the need for emergency surgery, periprocedural stroke, need for a permanent pacemaker (PPM) implant, paravalvular leak (PVL), more than mild residual gradient, and vascular complications.

Procedure

Procedures were performed using deep conscious sedation (DCS) or general anesthesia (GA). Transfemoral access was our first choice whenever possible. Vascular access was taken using a fluoroscopically guided roadmap taken from the opposite side femoral access. For a percutaneous TAVI procedure, we usually use 2 ProGlide suture devices for a 14-Fr sheath. During the procedure, targeted activated coagulation time was 250-300 ms and measured every 30 minutes. Balloon tip TPI was positioned preferably at the right ventricular apex with an aimed pacing rate leading to systolic blood pressure below 80 mmHg during valve deployment. Pigtail cine aortic root angiograms were taken initially in a co-planar view. Coronary protection was used in required cases beforehand. We prefer to cross the aortic valve using an Amplatzer left catheter with a straight-tip 0.035” Teflon wire and valve navigation over the 0.035” ES-Safari or Lunderquist wire. Valve deployment is preferred at a depth of 20% on the left ventricular side and 80% toward the aorta with a second Myval dark band at the aortic annulus position [7]. Post-deployment transthoracic echo was performed to check for residual gradient and PVL.

**Figure 1:**
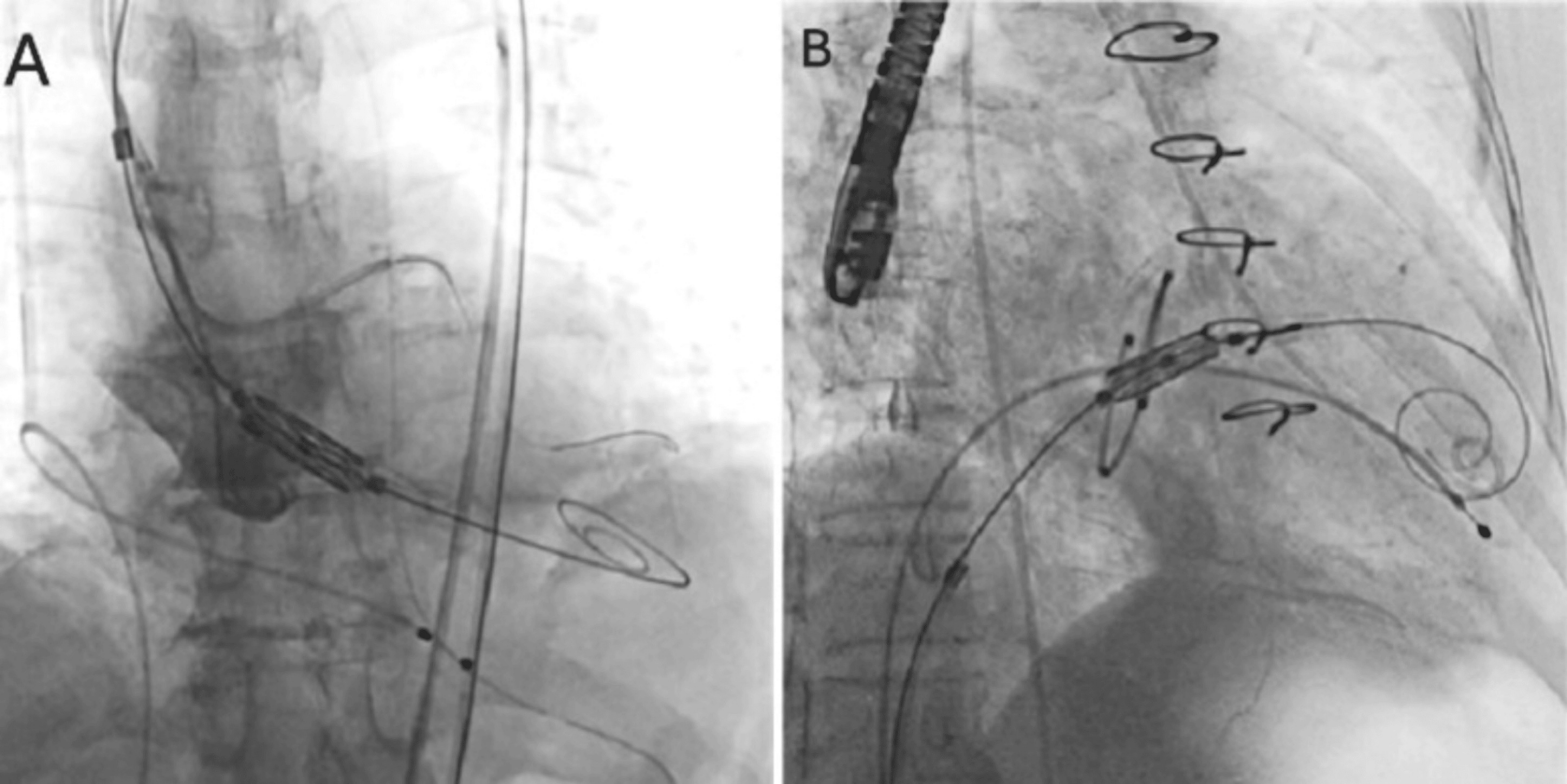
Myval deployment, at the position of A: aortic (aortic-ventricular deployment ratio 80:20) B: mitral (atrial-ventricular deployment ratio 20:80)

For the TMVR case, transfemoral venous access was taken, and the trans-septal puncture was done using the Brockenbrough needle (Medtronic) under transesophageal echocardiography (TEE) guidance in the superior and posterior portion of fossa-ovalis and 3-3.5 cm above the mitral annular plane. For the crossing of the mitral valve, a 5 Fr Judkins right catheter inside a flexible 8.5 Fr Agilis sheath (St Jude Medical, USA) with a straight-tip Teflon wire was used. Positioning was executed perpendicular to the plane of the mitral ring over a 0.035” ES-safari wire. The valve was deployed at a depth of 80% on the left ventricular side and 20% toward the left atrium (Figure 1) [14].

Hemostasis was achieved using percutaneous ProGlide suture devices for femoral arterial access and figure-of-eight sutures for femoral venous access. After removing the sheath, peripheral angiography was performed to evaluate the access site patency. Following a successful procedure, patients were given dual antiplatelet therapy consisting of 75 mg clopidogrel and 75 mg aspirin for six months, followed by a single antiplatelet therapy [15].

## Results

The mean age in our study was 75.87±7.5 years (mean±SD), with a minimum age of 64 years and a maximum of 90 years. Eight patients (53.3%) were female. All the patients were symptomatic and presented in NYHA functional class II to IV. A total of nine patients (60%) were presented in NYHA class IV. Based on the EuroSCORE-II score risk group, six patients (40%) were in the intermediate surgical risk group, and nine (60%) were in the high-risk group. The mean EuroSCORE-II score was 7.99±5.64% (mean±SD). In our TAVI cohort of 14 patients, all had symptomatic severe AS. A total of three (20%) patients had low-flow, low-gradient severe AS and associated severe left ventricular dysfunction (LVEF<30%). Baseline characteristics are presented in Table 1.

**Table 1:** Baseline demographic data Values are expressed as n (%) or mean±SD AS: aortic stenosis; AR: aortic regurgitation; MS: mitral stenosis; MR: mitral regurgitation

Baseline demographic data	Total N=15 (%) or mean±SD
Age, year	75.87±7.51
Min age	64 year
Max age	90 year
Sex	
Male	7 (46.67)
Female	8 (53.33)
EuroSCORE II predictive operative mortality	7.99±5.64%
Implant site calcification	
Mild	6 (42.86)
Moderate	3 (21.43)
Severe	5 (35.71)
CT measured area, mm^2 ^	625.29±388.70
Implant site stenosis	
Severe AS	14 (93.33)
Low flow low gradient severe AS	3 (20.00)
Moderate MS	1 (6.67)
Regurgitation (echo measured)	
Mild AR	5 (33.33)
Moderate AR	4 (26.67)
Severe MR	1 (6.67)

In our study, a total of 11 (73.3%) patients had a tricuspid aortic valve, and three (20%) patients had a bicuspid aortic valve (two patients were type 0 and one had type I-b bicuspid aortic valve). None of the patients had severe aortic regurgitation (AR), while four patients (26.67%) had moderate AR. In our TAVI cohort, five patients (33.3%) had severe aortic valve calcification according to the CT severity of aortic valve calcification score. Our patients' mean aortic valve calcification score was 625±388.70 (mean±SD) mm^2^. Although a selection of Myval size depends on multiple factors, it is primarily selected by CT-measured 3D aortic annulus area. In our study, two patients had a Myval implant of 20 mm, two had 21.5 mm, four had 23 mm, five had 24.5 mm, and one had an implant of 26 mm Myval at the aortic position. Details of the annulus and Myval size are presented in Table 2.

**Table 2:** Details of annulus and Myval size Values are expressed as n (%) or mean±SD. TAVI: transcatheter aortic valve implantation; TMViR: transcatheter mitral valve-in-ring

Details of annulus and Myval size	N=15 (%) or (mean±SD)
Procedure	
TAVI	14 (93.33)
TMViR	1 (6.67)
Myval implant site	
Mitral complete rigid ring	1 (6.67)
Native bicuspid (type 0) aortic valve	2 (13.33)
Native bicuspid (type Ib) aortic valve	1 (6.67)
Native tricuspid aortic valve	11 (73.33)
Implant site annulus area, mm^2^	390.20±74.49
Area derived diameter, mm	22.19±2.17
Myval size, mm	
20	2 (13.33)
21.5	2 (13.33)
23	5 (33.33)
24.5	5 (33.33)
26	1 (6.67)

The procedures were conducted under DCS unless GA was specifically necessary. All TAVI procedures were performed through the femoral route (one patient was approached via the left femoral artery, while the others were through the right femoral artery) with three (20%) cases requiring femoral arterial cut-down and the remaining 12 (80%) cases utilizing the percutaneous Seldinger technique, guided by an angiographic roadmap from the contralateral side. For 14 Fr arterial access, two ProGlide closure devices were used.

We generally avoid pre-dilation of the native valve, reserving it for situations where valve navigation could be difficult due to factors such as severe calcification, a horizontal aorta, or bicuspid valve anatomy. Pre-balloon was conducted in five of the TAVI patients. None of the patients experienced severe AR or hemodynamic instability following ballooning. We used coronary protection in case of low coronary heights (<10 mm from aortic annulus) and narrow sinuses of Valsalva patients with a heart team discussion for every patient. Coronary protection was required in four of our TAVI (28%) cases and a guide catheter with a guide extension was used beforehand, while none required coronary stenting post-valve deployment. One patient needed emergency coronary stenting under extracorporeal membrane oxygenation (ECMO) support before valve deployment as guide-induced dissection during coronary protection.

We did not routinely use embolic protection devices during the TAVI procedure. Valve deployment in TAVI patients was typically preferred at a depth of 20% on the left ventricular side and 80% toward the aorta in the co-planar view, with the second dark band at the aortic annulus. None of the patients experienced embolization or valve migration. Among the 11 TAVI patients who had the ProGlide device applied, two patients experienced a ProGlide failure (13.3%), which was managed by compression. None of the patients developed femoral hematoma and access site complications. One patient had a 14-Fr hydrophilic expandable Python sheath rupture during retrieval of the delivery system, which was managed by a change of sheath. The procedural data is shown in Table 3.

**Table 3:** Procedural consideration Values are expressed as n (%) or mean±SD DCS: deep conscious sedation; GA: general anesthesia; LCA: left coronary artery; RCA: right coronary artery

Procedural consideration	N=15 (%) or mean±SD
Anesthesia	
DCS	11 (73.33)
GA	4 (26.67)
Access site	
Right femoral artery	13 (86.67)
Right femoral vein	1 (6.67)
Left femoral artery	1 (6.67)
Diameter, mm	7.51±1.08
Approach	
Percutaneous	12 (80.00)
Arterial cut-down	3 (20.00)
LCA height, mm	12.80±3.44
RCA height, mm	15.91±2.65
Coronary protection required	4 (28.57)
Pre-balloon	
16*40 mm/mammoth	2 (13.33)
18*40 mm/mammoth	3 (20.00)
20*40 mm/mammoth	3 (20.00)
Post-balloon	
with extra 1 cc	2 (13.33)
with extra 2 cc	1 (6.67)
Complication	
ProGlide failure	2 (13.33)
Navigator balloon rupture; sheath tear	1 (6.67)

Our two patients had mild PVL; post-balloon was done in these cases with an extra 1-2 cc volume, and the PVL was decreased in both cases. One patient with bicuspid valve type 0 had a significant residual gradient (mean: 28 mmHg), so we did post-dilation with an extra 2 cc volume; after post-dilation, the mean gradient reduced by 4 mmHg, and we accepted the result. No patients had a central jet of significant AR.

In our Myval implant cases, none of the patients had valve migration and needed a second valve. The mean post-procedural hospital stay was 2.9 days. No patients had a periprocedural stroke or needed a PPM implant.

One patient underwent a Myval implant at the mitral position inside a complete rigid mitral ring (Edwards IMR Etlogix 24 mm ring). The patient presented with symptomatic moderate mitral stenosis and severe mitral regurgitation. The transeptal puncture was done at the fossa ovalis region under TEE guidance at a height of 3 cm from the mitral valve septal plane using a Brockenbrough needle (Medtronic). The septal dilation was done with 14- and 18-mm Mammoth balloons and the mitral ring was pre-dilated with a 20-mm Atlas-gold balloon. A 23 mm Myval was deployed at a depth of 80% on the left ventricular side and 20% toward the left atrium side. During deployment, the navigator balloon was ruptured at the end of balloon inflation, but the valve did not embolize or migrate. Post-deployment TEE showed no valve deformation or Neo-LVOT obstruction, but mild PVL was present. Post-ballooning was done with an extra 2 cc volume, which led to the disappearance of PVL. The mean gradient across the mitral valve was reduced from 9 mmHg to 3 mmHg, and there was no central jet of MR.

The 30-day survival rate among our Myval implant patients was 100%. All patients demonstrated improvement in their NYHA functional class postoperatively, at least by one grade. During a mean follow-up period of 22 months, two patients (14.3%) passed away. One patient succumbed to sudden cardiac death at home 50 months after the procedure, while another died three months post-procedure due to a community-acquired lung infection.

None of the patients experienced a stroke or myocardial infarction following valve deployment. However, one patient was diagnosed with a chronic subdural hematoma six months after the procedure, which was successfully treated surgically with no lasting neurological deficits. Additionally, one patient with chronic stable angina underwent percutaneous coronary revascularization of the left circumflex and right coronary arteries following the TAVI procedure without any complications. The complications and follow-up are shown in Table 4.

**Table 4:** Complications and follow-up Values are expressed as n (%) or mean±SD PVL: paravalvular leak; SCD: sudden cardiac death; SDH: subdural hematoma; LCX: left circumflex; RCA: right coronary artery

Complications and follow-up	N=15 (%) or mean±SD
Residual significant stenosis or regurgitation at the implant site	
Trace PVL	2 (13.33)
Residual mean gradient 24 mmHg	1 (6.67)
Residual mean gradient 3 mmHg	1 (6.67)
Follow-up period, months	22.00 ±21.42
Follow-up status	
Doing well	11 (73.33)
Died due to SCD after 53 months of the index procedure	1 (6.67)
Died due to lung infection after 3 months of the index procedure	1 (6.67)
Operated for chronic SDH after 3 months of the index procedure	1 (6.67)
PCI to LCx and RCA after 3 months of the index procedure	1 (6.67)

## Discussion

**Figure 2:**
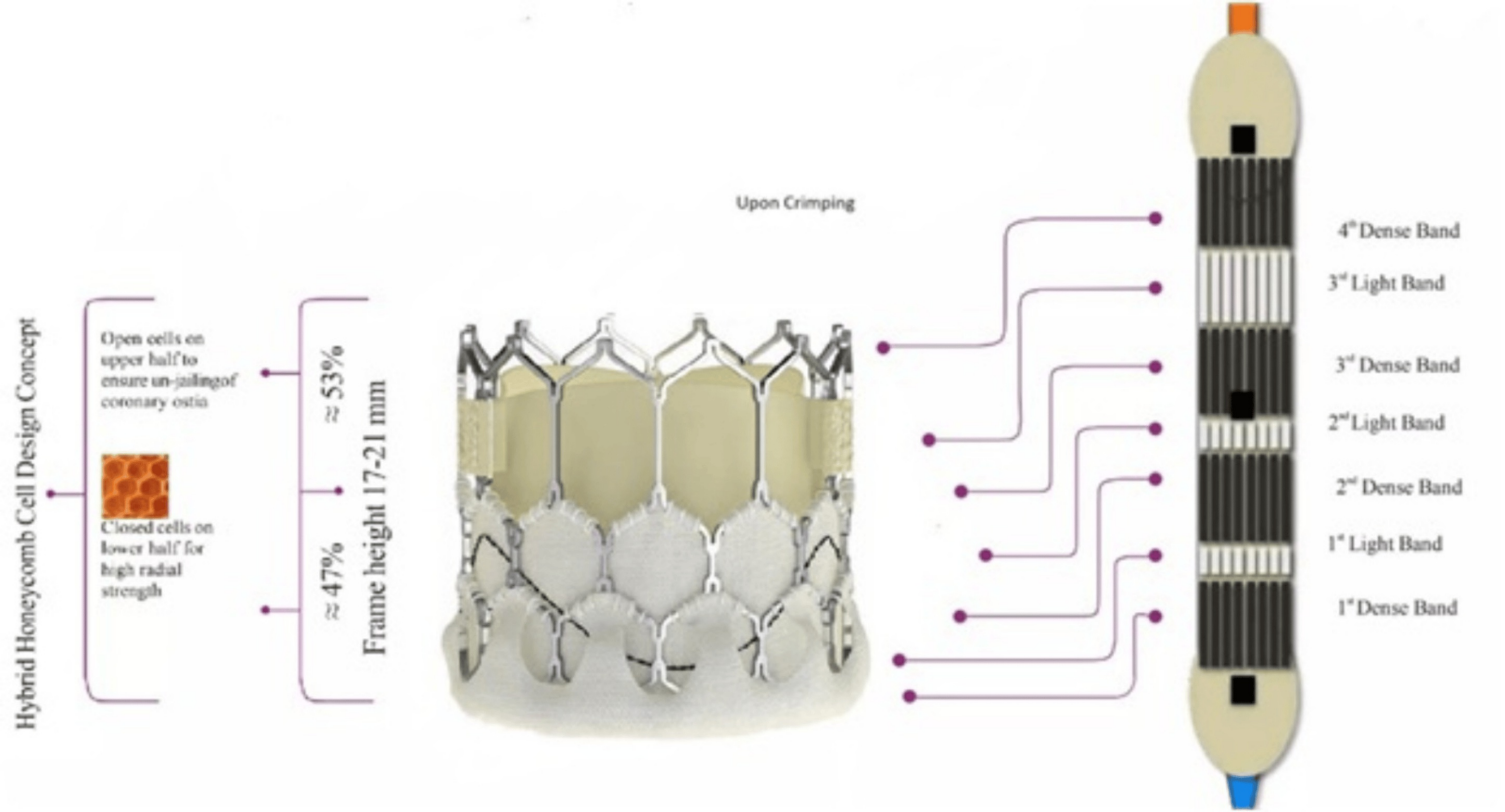
Myval: before and after crimping Credits: Meril Life Sciences, Vapi, India. Reproduced with permission from Meril Life Sciences.

**Figure 3:**
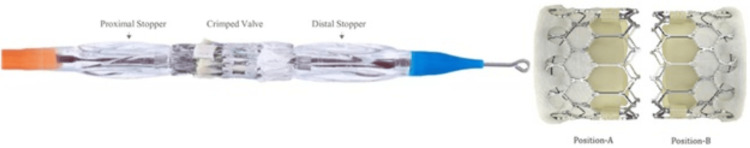
Crimping position of Myval on navigator balloon; A for mitral and B for aortic Credits: Meril Life Sciences, Vapi, India. Reproduced with permission from Meril Life Sciences.

The Myval, a balloon-expandable THV (Meril Lifesciences, India), is made of a nickel-cobalt alloy in a hexagonal honeycomb frame design with a 47% frame height of a closed-cell design (2 rows) to give radial strength and a 53% height of an open-cell design (1 row) to prevent the jailing of coronary ostia. The "closed cell" portion is covered externally with a polyethylene terephthalate sealing cuff to minimize the PVL. The valve leaflets comprise decellularized bovine pericardium tissue, crafted into a tri-leaflet valve. After crimping, the valve geometry results in alternating dark and light bands that are used for reference markers during the positioning and deployment of the valve across the annulus (Figures 2-3).

The available sizes start from 20 mm, with every 1.5 mm increment to 32 mm [16]. All crimped Myval are compatible with a 14-Fr hydrophilic expandable Python sheath that can permit complete retrieval of the valve from the patient. The delivery system allows flexion of the distal end up to more than 180 degrees, ensuring non-traumatic negotiation across the aortic arch and minimizing the risk of a periprocedural stroke. The balloon has proximal and distal internal inflation ports, resulting in dog-bone-shaped inflation and two counter-opposing soft stoppers that prevent the valve from dislodging during delivery. For inflation balloons, the saline:contrast ratio should be a minimum of 3:1, and 6 atm is the rated burst pressure. For pre-dilation, a 9-Fr compatible Mammoth over-the-wire non-compliant balloon is available in 14 to 30 mm in various sizes with 40 mm lengths also developed by Meril Life Sciences [16].

From March 2019 to August 2024, in our tertiary care center, we deployed 14 Myval implants at the native aortic valve position and one at a mitral position within the complete rigid mitral ring. The mean age in our study was 75.87±7.51 years (mean±SD), and eight (53.3%) patients were female. The mean age was comparable to the Myval-1 study, while the female population was more compared to the male.

The mean EuroSCORE-II score was 7.99±4.21%. All patients were symptomatic with NYHA functional class II-IV, and 60% were presented in NYHA class IV. In the Myval-1 study, intermediate and high-risk surgical patients were included, and mean STS scores of 6.4±1.8, and 16.7% were in functional class IV [7].

No patients had embolization or migration of the valve and did not need a second valve in any of the cases. None of the patients developed periprocedural stroke. In our study, one of the patients had coronary obstruction during coronary protection and underwent bailout coronary stenting over ECMO support. In our study, none of the patients needed PPM implantation periprocedurally and over a 22-month mean follow-up period. The PPM implant rate after SAPIEN 3 was 12.5% in PARTNER 2 registries [17], while in the Myval-1 study, none of the patients needed a PPM implant [7].

Previous studies with SAPIEN 3 have shown that PVL is associated with a poor prognosis after TAVI [18,19]. In total, two of our TAVI patients had mild PVL after valve deployment, and in these cases, post-ballooning with extra volume led to a decrease in PVL in both cases. Our results were comparable with the Myval-1 study [7].

In our TMVR case, we had a balloon rupture during deployment, which occurred during the last part of balloon inflation, but the valve did not embolize or migrate. No significant neo-LVOT gradient has occurred.

There were no procedural or in-hospital deaths among our Myval patients, and the 30-day survival rate was 100%. Over a mean follow-up period of 22 months, two patients (14.2%) died; one due to community-acquired pneumonia and the other from sudden cardiac death. By comparison, the Myval-1 study reported a 12-month mortality rate of 13.3%. All patients in our cohort showed postoperative improvement in the NYHA functional class [7].

A total of three patients were re-admitted after the procedure, one due to community-acquired pneumonia after 90 days and expired for the same reason, while another patient was found to have chronic subdural hematoma six months after the procedure, which was managed surgically without residual neurological deficit. One of the patients required coronary revascularization for refractory stable angina three months after the index procedure and it was successfully done without complication.

In our study, the ProGlide failure rate was 13.3%, and failure occurred during knot tightening due to thread break, which is well managed by compression. No femoral access site complications occurred in our patients. One patient had a sheath tear during retrieval of the delivery system, and it was managed with the exchange of another sheath.

Limitations

The major limitations of this study are the limited number of patients and the short follow-up period.

## Conclusions

Our observations indicate that the Myval device shows a promising efficacy profile and good procedural success rates with encouraging early outcomes in transcatheter valve procedures. While there are some concerns about balloon rupture occurring below the rated burst pressure and the integrity of the sheath, it remains a cost-effective choice for balloon-expandable transcatheter heart valve option for both aortic and mitral interventions based on our limited experience.
